# Epidemiology and treatment patterns of urachal remnants in adult patients with omphalitis: a nationwide claims-based study in Japan

**DOI:** 10.1007/s00595-025-03151-6

**Published:** 2025-10-15

**Authors:** Ryota Tokunaga, Takenori Yamauchi, Hiroki Den, Shunsuke Omotaka, Suguru Ogihara, Masayuki Isozaki, Takahiro Hobo, Noboru Yokoyama, Haruhiro Inoue, Akatsuki Kokaze

**Affiliations:** 1 Department of Hygiene and Public Health and Preventive Medicine, Showa Medical University Graduate School of Medicine, 1-5-8 Hatanodai, Shinagawa-ku, Tokyo, Japan; 2 Digestive Disease Center, Showa Medical University, Koto Toyosu Hospital, 5-1-38, Koto-ku, Tokyo, Japan

**Keywords:** Omphalitis, Urachal remnants, Epidemiology, Claims database

## Abstract

**Purpose:**

Urachal remnants are common in neonates and can persist into adulthood. However, their epidemiology in adults remains poorly characterized. We aimed to investigate the prevalence and clinical features of urachal remnants in adult Japanese patients with omphalitis using a nationwide claims database.

**Methods:**

We analyzed data from the Japan Medical Data Center Claims Database (2005–2023) to identify patients  ≥ 15 years of age who were diagnosed with omphalitis. The prevalence of urachal remnants, associated diagnoses, surgical interventions, and time to surgery was examined, focusing on sex differences and age distribution.

**Results:**

Of the 11,477 patients with omphalitis, 1836 (16.0%) had urachal remnants, with a male-to-female ratio of 2.53:1. The prevalence peaked in males of 20–34 years of age, exceeding 30%. Surgical intervention was performed in 39.7% of the cases, with a median time to surgery of 2 months. The number of laparoscopic procedures increased after 2014 and surpassed that of open surgeries by 2018. One case of urachal cancer (0.054%) was also identified.

**Conclusion:**

Urachal remnants are relatively common in adults with omphalitis, particularly in young males, and usually require surgery. Given the high prevalence and risk of recurrence, early imaging should be considered in adult omphalitis cases to support a timely diagnosis and intervention.

## Introduction

Omphalitis is a common condition in outpatient surgical practice. It is an infection or inflammation of the umbilicus and adjacent tissues, particularly in newborns. Reports of omphalitis in adults are rare and only a few case studies are available [1]. Omphalitis is caused by anatomical abnormalities and infections resulting from foreign bodies in the umbilicus such as navel piercings [1]. Among non-foreign body-related causes, urachal abnormalities are particularly significant, with omphalitis secondary to urachal remnants being the most frequently reported cause. Urachal remnants are relatively rare anomalies resulting from the persistence of the urachus [2], which is a tubular structure that extends from the umbilicus to the bladder apex [3]. These remnants are present in approximately 1.03% of children [4, 5]. However, urachal remnant-induced omphalitis is rare in adults. Moreover, in the 2023 National Clinical Database, urachal resection was only described in pediatric surgery, and no adult operations were reported [6]. This discrepancy may be attributed to the presence of two types of urachal abnormalities, childhood- and adult-onset, each with unique pathogenic mechanisms [7]. Childhood-onset type, predominantly in newborns and children, results from a persistently patent urachal cavity, allowing free urinary discharge from the umbilicus. In contrast, the adult-onset type occurs when an urachus that is closed at birth reopens later in life. Consequently, adult-onset type is more frequent in individuals of 20–40 years of age [7].

While current epidemiological studies on urachal remnants have primarily focused on childhood-onset cases in newborns, large-scale epidemiological investigations on adult-onset cases are lacking. Most recent studies on relatively rare conditions have used large-scale insurance claims data [8–12]. In the present study, we focused on adult-onset urachal abnormalities to determine the proportion of patients with urachal remnants among patients diagnosed with omphalitis, assess the percentage of cases requiring surgical intervention, and examine the associated timeline. In addition, we aimed to clarify the extent to which healthcare providers should consider the possibility of urachal remnants when evaluating patients with omphalitis in an outpatient surgical setting.

## Methods

### Data source

This retrospective observational study used data from the Japan Medical Data Center (JMDC) Claims Database, a nationwide repository of Japanese insurance claims. The database contains health insurance claims from individuals enrolled in employment-based insurance systems in Japan, composed of 17,656,319 individuals (9,045,717 males and 8,610,602 females) managed between 2005 and August 2023[13]. The data included patient registration details, information on medical institutions, diagnoses, procedures, medications, medical materials, annual health examinations, and visit-related costs [14]. In addition, the database provides information on age, sex, drug prescriptions, and diagnostic codes based on the International Classification of Diseases, 10th revision [15–17]. These data enable the analysis of medical service utilization across diverse healthcare settings, including large medical centers, small- and medium-sized hospitals, and clinics with inpatient beds [12]. All data were anonymized and personal identifiers were removed to ensure patient privacy. This study was approved by the Ethics Committee for Research Involving Human Subjects at Showa University (approval number: 2024–247-A). This study conforms to the provisions of the Declaration of Helsinki.

### Definition of omphalitis and urachal remnants

Omphalitis is defined as an infection of the umbilicus and surrounding tissues, characterized by symptoms such as redness, swelling, warmth, pain, and discharge. In this study, patients were classified as having omphalitis if they tested positive for any of the diagnoses listed in Table 1. Urachal remnants were defined as pathological conditions emerging from persistent urachal structures, including patent urachus. Patients were classified as having urachal remnants if they tested positive for any of the diagnoses summarized in Table 2.

**Table 1:** Omphalitis types and corresponding Japanese standard disease codes and ICD-10 codes

Name of omphalitis	Japanese standard disease codes	ICD-10 codes
Periumbilical pain	7890022	R103
Infraumbilical pain	7890021	R103
Umbilical carbuncle	8833978	L022
Umbilical fistula	8833987	Q898
Umbilicovesical fistula	8833981	Q648
Persistent umbilical pain	8835218	R103
Periumbilical cellulitis	6869050	L089
Umbilical mass	8833975	R190
Neonatal omphalitis	7714001	P38-
Umbilical abscess	8845292	L022
Recurrent umbilical pain	8839039	R103
Umbilical furuncle	8833976	L022
Umbilical phlegmon	8833977	L033
Neonatal umbilical hemorrhage	8835025	P519

*ICD* International statistical classification of diseases and related health problems

**Table 2:** Urachal remnant types and corresponding Japanese standard disease codes and ICD-10 codes

Name of omphalitis	Japanese standard disease codes	ICD-10 codes
Urachal sinus	8849430	Q644
Urachal diverticulum of the bladder	8849429	Q644
Urachal remnant	8849427	Q644
Urachal abscess	8845026	N308
Urachal cyst	8838559	Q644
Urachal anomaly	8838557	Q644
Urachal malformation	8838560	Q644
Urachal carcinoma	1887002	C677
Patent urachus	8849428	Q644

*ICD* International statistical classification of diseases and related health problems

### Identification of patients with urachal remnants and subsequent surgical cases among patients with omphalitis

#### Patients with omphalitis

This study included patients who met the following criteria: omphalitis onset at (1) ≥ 15 years of age and (2) ≥ 3 months after observation commenced. Criterion 1 was established to focus on adult patients as individuals < 15 years of age who received care from pediatricians. Criterion 2 was included to exclude patients who may have been undergoing treatment before the observation window, as this study aimed to capture new outpatient cases. Additionally, patients who had omphalitis-related visits within the first two months of the observation period were excluded to further minimize the inclusion of ongoing or pre-existing cases.

#### Patients diagnosed with urachal remnants

Among the patients diagnosed with omphalitis and urachal remnants, those without any flagged diagnostic uncertainty were identified as confirmed cases of both conditions.

#### Patients undergoing urachal excision surgery

**Fig. 1:**
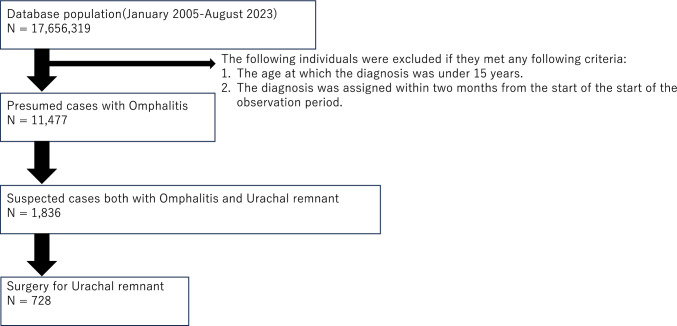
Participant selection flowchart. Flowchart of study patients using the JMDC database (January 2005 to August 2023). Among the patients enrolled in the database, we extracted patients  ≥ 15 years of age who were diagnosed with omphalitis at least 3 months after the observation commenced. Subsequently, we selected patients diagnosed with residual ureteral duct disease. Finally, we extracted patients who underwent surgery. *JMDC* Japan Medical Data Center

Patients who underwent any of the procedures listed in Table 3 were classified as having undergone urachal excision. The complete process flow is shown in Fig. 1.

**Table 3:** Urachal excision types and corresponding Japanese standard procedure codes

Name of urachal excision	Japanese standard procedure code
Urachal excision	150201010
Laparoscopic urachal excision	150379510

#### Extracted data points

The extracted data included birth month and year, sex, insurance enrollment month and year, date of the omphalitis diagnosis, date of the urachal remnant diagnosis, date of urachal excision surgery, and type of surgical procedure (See Table 4).

**Table 4:** Relationship between the presence or absence of urachal remnant and test items. In Japan, alcohol intake was investigated based on 1 cup of sake, so the average alcohol content of 1 cup of sake is displayed in the table as 20 g

		No urachal remnant	Urachal remnant	Significant difference
Waist circumference (cm)		83.3 ± 12.2	78.5 ± 10.1	+
Systolic blood pressure (mmHg)	On medication	129.6 ± 16.4	131.9 ± 13.7	
	No medication	118.1 ± 15.4	115.4 ± 13.7	+
Diastolic blood pressure (mmHg)	On medication	80.6 ± 10.4	80.8 ± 7.9	
	No medication	72.5 ± 11.6	70.5 ± 10.5	+
Triglycerides(mg/dl)		101.5 ± 70.7	93.3 ± 57.5	+
HDL-cholesterol(mg/dl)		63.2 ± 16.4	62.3 ± 15.2	+
Fasting blood glucose(mg/dl)		94.0 ± 15.7	90.5 ± 10.7	+
HbA1c (%)		5.5 ± 0.6	5.3 ± 0.4	+
BMI (kg/m^2^)		23.6 ± 4.6	21.8 ± 3.6	+
Smoking (%)	Smoking	21.6	29.4	
	Non-smoking	78.4	70.6	
Drinking frequency(%)	Daily	17.8	19.1	
	Occasionally	34.9	40.9	
	Rarely	47.3	39.5	
Alcohol consumption (%)	< 20 g/day	56.6	49.1	
	≥ 20 g/day, < 40 g/day	27.9	33.5	
	≥ 40 g/day, < 60 g/day	11.3	12.2	
	≥ 60 g/day	4.2	5.1	

#### Analyzed variables

The analyzed variables included the distribution of age and sex for the omphalitis diagnosis, urachal remnant diagnosis, and urachal excision surgery. Additionally, we examined the number and proportion of patients diagnosed with urachal remnants among those with omphalitis stratified by age and sex. Furthermore, the analysis included age and the number of patients who underwent surgery among patients diagnosed with urachal remnant-related conditions. All analyses were limited to patients  ≥ 15 years of age, including only records in which the time between the patient’s birth month and their earliest omphalitis-related diagnosis exceeded 14 years and 10 months. Data were extracted using PostgreSQL (ver. 16.0). Data aggregation was performed using Microsoft Excel (2019).

## Results

Among the patients ≥ 15 years of age, 11,477 cases of omphalitis were identified (6293 males and 5184 females), with a male-to-female ratio of 1.21:1, indicating a slight male predominance. Of these, 1836 patients (1386 males and 450 females) were diagnosed with urachal remnants, representing 16% of all omphalitis cases. When analyzed by sex, 22% of the male patients with omphalitis and 8.7% of the female patients had urachal remnants, indicating an evident male predominance.

**Fig. 2:**
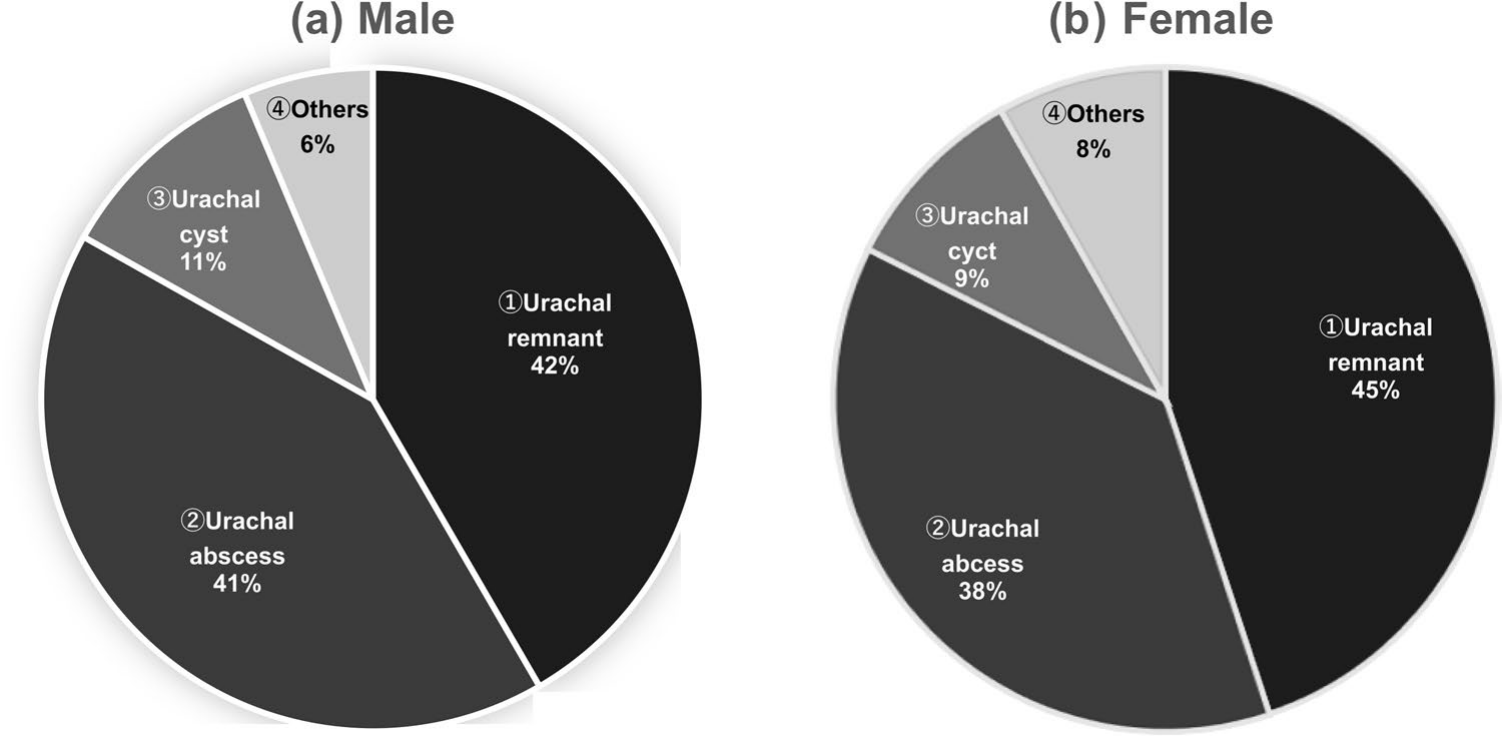
Rate of the diagnoses of urachal remnant disease by sex. **a** Male: 1. Urachal remnant, 2. Urachal abscess, 3. Urachal cyst, 4. Other. **b** Female: 1. Urachal remnant, 2. Urachal abscess, 3. Urachal cyst, 4. Other. No sex differences were noted

**Fig. 3:**
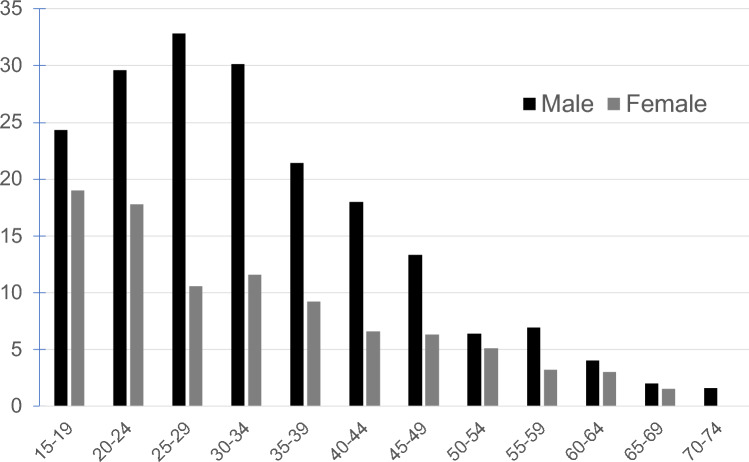
Proportion of patients with urachal remnants among those with omphalitis, stratified by sex and age group. Urachal remnants were more common in patients 15–39 years of age. In particular, men of 20–39 years of age had a significantly higher incidence than women

Among patients with omphalitis, the most common urachal-related diagnoses in both sexes, in descending order, were urachal remnant > urachal abscess > urachal cyst (Fig. 2). An age-specific analysis revealed that urachal remnants were most prevalent among patients 15–39 years of age. For male patients, the distribution was as follows: ages 20–24, 29.6%; 25–29, 32.8%; 30–34, 30.1%; and 35–39, 21.4%. The percentages of female patients were 17.8% (20–24 years), 10.6% (25–29 years), 11.6% (30–34 years), and 9.2% (35–39 years) (Fig. 3). Urachal remnants were more prevalent in males than in females, particularly in the 20–39-year age group. Notably, among the 1836 patients with urachal remnants, one case of urachal cancer was identified (0.054%).

We investigated the relationship between the presence or absence of urachal remnants and the test items. Data were compared with six items related to metabolic syndrome: waist circumference, blood pressure (with or without antihypertensive drugs), triglycerides, high-density lipoprotein cholesterol, fasting blood glucose, and HbA1c. In addition, body mass index, smoking, opportunity to drink, and the amount of alcohol consumed were also tested. The results showed significant differences in all items related to metabolic syndrome, except for blood pressure, among people taking antihypertensive drugs. Smoking and alcohol consumption showed no significant difference between the presence and absence of urachal remnants.

Of the 11,477 patients with omphalitis, 728 underwent surgery, accounting for 6.1% of the total (male: 570 of 6293, 9.06%; female: 158 of 5184, 3.04%). Furthermore, 39.7% (728 of 1836) of the patients diagnosed with urachal remnant disease underwent surgery (males: 570 of 1386 (41.0%); females: 158 of 450 [35.1%]).

**Fig. 4:**
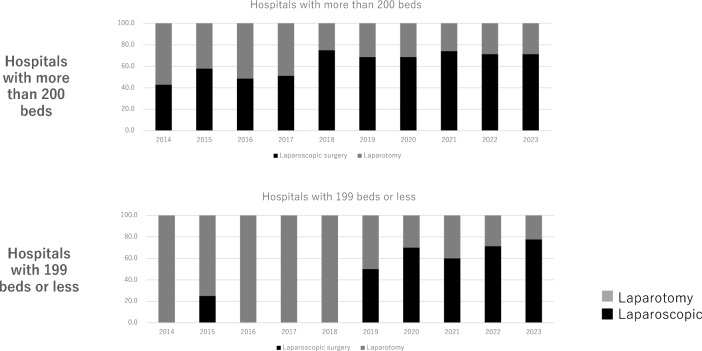
Annual trends in surgical procedures for urachal resection, compared by number of hospital beds. Urachal excision surgery was performed in Japan in 2006. The number of laparoscopic urachal excisions has gradually increased since it was covered by insurance in 2014. In the beginning, most surgeries were performed in hospitals with more than 200 beds; however, since 2019, laparoscopic surgeries have been increasing in hospitals with less than 200 beds. (Prior to 2013, laparoscopic surgery was not covered by insurance and could not be accurately evaluated; therefore, only data from 2014 onwards were used.)

**Fig. 5:**
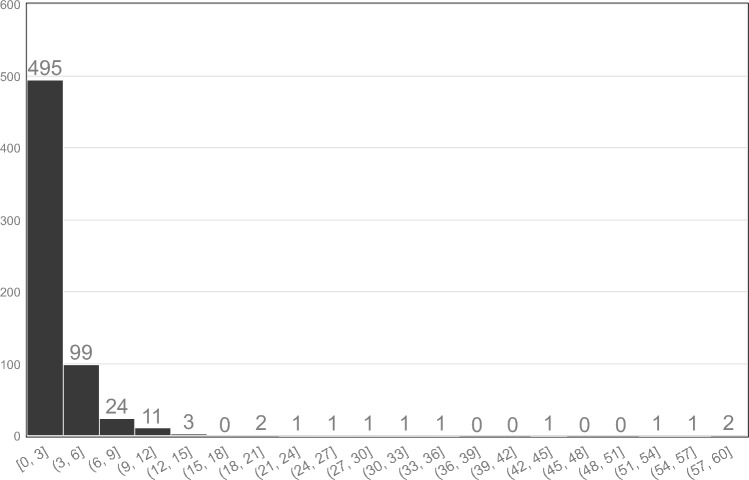
Duration (in months) from the diagnosis of urachal remnant to surgery. The time interval between the diagnosis of urachal remnant and excision surgery ranged from 0 to 60 months, with a median of 2 months

Records of urachal excision surgery date back to 2006, with the first laparoscopic procedure being performed in 2014. Since 2018, the total number of surgeries performed has increased annually, with laparoscopic surgeries being significantly more frequent than open procedures. In addition, laparoscopic surgeries have constituted > 40% of all procedures since 2014 in hospitals with ≥ 200 beds. In hospitals with < 200 beds, the frequency of laparoscopic surgeries has steadily increased since 2019 (Fig. 4). The time interval between the urachal remnant diagnosis and excision surgery ranged from 0 to 60 months (median, 2 months) (Fig. 5).

## Discussion

In this study, we conducted an epidemiological investigation of omphalitis and urachal remnants in Japan using data from the JMDC Claims Database from 2005 to August 2023. We specifically examined the prevalence of urachal remnants among patients  ≥ 15 years of age who presented with symptoms of omphalitis.

Patients with urachal remnants have symptoms other than omphalitis. In cases of infection or bleeding on the bladder side of the ureteral duct, blood in the urine or cystitis, and infection in the ureteral duct that is not in contact with the umbilical cord or bladder, lower abdominal pain may occur. However, owing to the nonspecific nature of tract infections and lower abdominal pain, which can be associated with various conditions and departments (e.g., urology), we selected only patients with omphalitis symptoms using insurance claims data to improve the specificity of our study.

While urachal remnants occur in 1.03–1.63% of children  < 15 years of age [4, 5], they are found in only 0.063% of adults [18–20]. However, among patients with symptoms of omphalitis, the prevalence of urachal remnants was notably high (16.0%). In male patients, the prevalence was 22.0%, highlighting a marked sex difference. These findings underscore the significance of age at onset and sex-based differences.

The median umbilical ligament, which connects the bladder to the umbilicus, is a remnant of the embryonic urachus that closes during early fetal development. Urachal remnants occur when closure is incomplete [21]. When these remnants are accompanied by symptoms, such as omphalitis, the condition is termed a urachal remnant disease [21, 22]. Given this developmental mechanism, remnant urachal disease is most common in newborns and children [1]. However, adult cases have also been reported, particularly in young adults [7, 23, 24]. This can be attributed to the existence of childhood- and adult-onset urachal remnant disease [23]. Childhood-onset type results from the persistence of a patent urachal cavity. As such abnormalities are discovered and treated at birth, childhood-onset urachal disease is rare in adults [7]. These cases usually involve anomalies of the urinary system and therefore require careful examination and testing [7].

Conversely, the adult-onset type occurs when a previously closed urachus reopens later in life, typically between 20 and 40 years of age. The urachal remnant cases identified in patients  ≥ 15 years of age in our study were primarily considered the adult-onset type. Urachal remnants without infections are usually asymptomatic and are discovered incidentally during imaging [4, 24]. However, symptomatic patients presenting with omphalitis or abdominal pain require medication or procedures, and surgery may be necessary if outpatient treatment is insufficient. Awareness of urachal remnants as a potential cause of omphalitis facilitates prompt diagnostic imaging and timely surgical intervention. This is particularly relevant for male patients, in whom more than one in five cases involves urachal remnants, making diagnostic testing crucial. Moreover, in the peak age range of adult-onset urachal remnant disease (20–34 years), the prevalence of omphalitis in males was 30%. Although urachal remnants were also present in females in this age group, the sex disparity was striking (Fig. 3). Various hypotheses regarding this difference have been proposed, including anatomical differences, hormonal influences, and genetic predispositions. However, the exact cause remains unclear and warrants further investigation.

Our study showed that 39.7% of patients  ≥ 15 years of age with urachal remnant disease (728 of 1836 patients) underwent surgery, indicating that 2 of 5 patients required surgical intervention. Several studies have explored the treatment strategies for childhood-onset urachal remnants. Nogueras-Ocaña et al. reported spontaneous regression in 8 of 13 patients (61.5%) [25], while Galati et al. reported spontaneous regression in 80% of patients  < 6 months of age [26]. Childhood-onset urachal remnant disease caused by incomplete closure during fetal development may resolve spontaneously [20] and usually improves without surgery [25]. However, the adult-onset type, which results from the reopening of a previously closed urachus, is less likely to resolve spontaneously [25]. Moreover, in most cases, urachal remnant-associated omphalitis cannot be definitively treated with outpatient care alone, owing to persistent structural abnormalities, with a reported recurrence rate of 30% [27]. Consequently, surgical treatment is recommended due to the risk of recurrence [25, 28].

The potential for malignant transformation is another factor that influences the decision to perform surgical intervention in urachal remnant cases [27]. Urachal cancer occurs in approximately 1 in 550,000–5.5 million individuals [29, 30]. However, > 80% of cases are diagnosed at Sheldon Stage ≥ III [31]. Furthermore, owing to the challenges of early detection, the prognosis remains poor, with a 5-year survival rate of 6.5–10% [29]. In our study, only one case of urachal cancer was identified among 1836 patients with urachal remnants, indicating that the frequency of urachal cancer in this population was significantly higher than that in the general population.

Moreover, we found that the median time to surgery was relatively short (two months). Of the two onset types, adult-onset urachal remnant disease may make surgical intervention more acceptable to both patients and clinicians. Given its pathogenesis, spontaneous resolution is less likely and prolonged and recurrent infections are common [22], leading to short observation periods before surgery [2, 20].

Patients who did not undergo surgery underwent outpatient treatment. Treatment methods included cleaning and disinfection of the wound, intravenous infusion and oral administration of antibiotics, and incision and drainage. Some patients were treated in the hospital, rather than on an outpatient basis. A few patients who did not undergo surgery experienced recurrence; however, most of the recurrent cases emerged after 1 year. Surgery is usually performed for recurrence within 1 year. Nevertheless, if the time to recurrence is long, patients may prefer to undergo follow-up.

Notably, surgical techniques have evolved from open to laparoscopic procedures. Although the surgeries in our dataset began in 2006, laparoscopic procedures have been performed since 1992. Following the approval of insurance coverage for laparoscopic urachal surgery in Japan in 2014 [29], the number of laparoscopic procedures has steadily increased [32], surpassing that of open surgeries after 2018. Furthermore, single-incision laparoendoscopic single-site surgery has garnered considerable attention [33, 34]. In terms of hospital size, there was a lag of approximately 5 years between the adoption of laparoscopic surgery in large hospitals (≥ 200 beds) and small- and medium-sized hospitals (< 199 beds). This suggests that adopting new surgical techniques across different hospital settings requires approximately 5 years.

This epidemiological study revealed a high prevalence of urachal remnants in patients with omphalitis, with a substantial proportion requiring surgical intervention. Our findings suggest that when treating recurrent or prolonged omphalitis in an outpatient setting, clinicians should consider the possibility of urachal remnants and proactively pursue imaging studies. Young individuals, particularly males 10–40 years of age, are more likely to have residual ureters. Therefore, further testing was required.

Additionally, patient characteristics may predict urachal remnants. Our study showed that patients with urachal remnants had better outcomes than those without urachal remnants, exhibiting most items in the metabolic syndrome evaluation criteria. This suggests that the presence of omphalitis symptoms in patients without abnormalities in lifestyle or health status (such as susceptibility to infections due to obesity or diabetes) is caused by ureteral remnants.

For less invasive screening, abdominal ultrasound is recommended [32], whereas more definitive assessments, such as abdominal computed tomography or magnetic resonance imaging, may be necessary to confirm the presence of urachal remnants [32].

We included only patients  ≥ 15 years of age because there is no standardized distinction between children and adults in Japan. Individuals  > 15 years of age are treated as adults and are likely to make their first visit to an adult outpatient department. Therefore, we defined adults in our study as those  > 15 years of age. Data on urachal excision surgery were first recorded in Japan in 2006. However, owing to the nature of insurance data, confirming the number of laparoscopic surgeries before 2014 is challenging, as laparoscopic surgeries were not covered by insurance. Therefore, we did not compare open and laparoscopic surgeries performed before insurance coverage. Our case identification relied on diagnostic codes for omphalitis and urachal remnant diseases. While these codes are entered by physicians during patient evaluation, they do not guarantee clinical accuracy, which is a common limitation of large-scale claims database research. Additionally, because “omphalitis” is not a standardized diagnosis code, some adult cases may have been recorded as “neonatal omphalitis,” potentially capturing recurrent rather than initial adult-onset cases. This coding ambiguity may introduce minor inaccuracies. However, as diagnostic codes are required to justify medical procedures and our study included all diagnoses related to the umbilicus, we believe that the risk of underestimating omphalitis cases was minimal. The database primarily included information from individuals enrolled in employment-based insurance programs, resulting in limited data for patients  > 70 years of age. Nonetheless, as omphalitis predominantly affects younger individuals, this age-related gap likely has a minimal impact on our conclusions. Regarding the treatment procedures, some surgical records may have been missed if they were not covered by insurance, as urachal remnant treatment is a relatively recent development. Despite this, the consistent trend toward minimally invasive techniques suggests that our data capture a broader trajectory of surgical evolution. Finally, while only one case of urachal cancer was identified in our dataset, it is important to note that our study focused on patients with omphalitis and did not provide a comprehensive estimate of the urachal cancer prevalence among all patients with urachal remnants. Future studies should aim to determine the true incidence of urachal cancer in this population.

Our epidemiological study of urachal remnants among patients  ≥ 15 years of age presenting with omphalitis symptoms highlights the potential significance of early imaging in guiding treatment strategies for adults with refractory or recurrent omphalitis given the high prevalence of urachal remnants in this population.
